# Rapamycin efficiently promotes cardiac differentiation of mouse embryonic stem cells

**DOI:** 10.1042/BSR20160552

**Published:** 2017-06-07

**Authors:** Qin Lu, Yinan Liu, Yang Wang, Weiping Wang, Zhe Yang, Tao Li, Yuyao Tian, Ping Chen, Kangtao Ma, Zhuqing Jia, Chunyan Zhou

**Affiliations:** 1 Department of Biochemistry and Molecular Biology, School of Basic Medical Sciences, Beijing Key Laboratory of Protein Posttranslational Modifications and Cell Function, Key Laboratory of Molecular Cardiovascular Sciences, Ministry of Education of China, Peking University, Beijing 100083, P.R. China; 2 Department of Biology, College of Chemistry and Life Sciences, Zhejiang Normal University, 688 Yingbin Road, Jinhua, Zhejiang 321004, China; 3 Department of Immunology, College of Medicine, Hunan Normal University, Changsha 410081, China; 4 Department of Cell Biology, Stem Cell Research Center, School of Basic Medical Sciences, Peking University, Haidian District, Beijing 100083, P.R. China

**Keywords:** cardiomyocytes, embryonic stem cells, mammalian target of rapamycin (mTOR), rapamycin

## Abstract

To investigate the effects of rapamycin on cardiac differentiation, murine embryonic stem cells (ESCs) were induced into cardiomyocytes by 10^−4^ M ascorbic acid (AA), 20 nM rapamycin alone or 0.01% solvent DMSO. We found that rapamycin alone was insufficient to initiate cardiomyogenesis. Then, the ESCs were treated with AA and rapamycin (20 nM) or AA and DMSO (0.01%) as a control. Compared with control, mouse ESCs (mESCs) treated with rapamycin (20 nM) and AA yielded a significantly higher percentage of cardiomyocytes, as confirmed by the percentage of beating embryonic bodies (EBs), the immunofluorescence and FACS analysis. Rapamycin significantly increased the expression of a panel of cardiac markers including *Gata*4, α-*Mhc*, β-*Mhc*, and *Tnnt*2. Additionally, rapamycin enhanced the expression of mesodermal and cardiac transcription factors such as *Mesp*1, *Brachyury T, Eomes, Isl*1*, Gata*4*, Nkx*2.5*, Tbx*5, *and Mef2c.* Mechanistic studies showed that rapamycin inhibits Wnt/β-catenin and Notch signaling but promotes the expression of fibroblast growth factor (*Fgf*8), *Fgf*10, and *Nodal* at early stage, and bone morphogenetic protein 2 (*Bmp* 2) at later stages. Sequential treatment of rapamycin showed that rapamycin promotes cardiac differentiation at the early and later stages. Interestingly, another mammalian target of rapamycin (mTOR) inhibitor Ku0063794 (1 µM) had similar effects on cardiomyogenesis. In conclusion, our results highlight a practical approach to generate cardiomyocytes from mESCs by rapamycin.

## Introduction

Cardiomyocytes derived from stem cells can survive and form stable intracardiac grafts to improve damaged cardiac function in animal models, thereby heralding the dawn of cell replacement for severe heart diseases. In the past decades, multiple cells, such as embryonic stem cells (ESCs), induced pluripotent stem cells (iPSCs) and mesenchymal stem cells (MSCs), were applied to repair damaged cardiac function [1–4]. Nevertheless, variable and insufficient cardiac differentiation efficiency impedes the application of cell therapy.

The supplement of various growth factors (BMP (bone morphogenetic protein)) BMP2, BMP4, activin A, bFGF (fibroblast growth factor)-2 (FGF2), FGF10, and Wnt3a (Wnt family member 3A)) during cardiac differentiation has been found to improve the induction efficiency of spontaneously beating cardiomyocytes [5–7], but the expensive costs limit large-scale utilization of these protein reagents. Accordingly, small molecule cocktails targetting crucial signaling pathways in cardiac differentiation are feasible to massively generate differentiated cardiac cells.

The mammalian target of rapamycin (mTOR) kinase is an atypical serine/threonine kinase and belongs to the family of phosphoinositide 3-kinases. mTOR exerts its main functions by forming two complexes: mTOR complex 1 (mTORC1) and mTOR complex 2 (mTORC2). Rapamycin and its analogs bind to the cytosolic FK506-binding protein (12 kDa) of mTORC1, thereby forming a protein complex that only targets a specific domain of the mTOR protein, when it is a part of mTORC1 [8]. As a consequence of rapamycin 12-kDa FK506-binding protein’s binding, mTORC1 activity is strongly inhibited [9]. Conversely, mTORC2 is relatively insensitive to rapamycin, although it has been reported that prolonged treatment with rapamycin inhibits mTORC2 assembly [10].

Upstream of mTOR, multiple signals exist, including insulin signaling through PI3K and AKT, energy signaling and stress response signaling through AMP-activated protein kinase(AMPK). These signals converge in the tuberous sclerosis complexes(TSC1–TSC2), which serves as a GTPase exchange factor for Rheb, whereas Rheb in GTP-bound form activates mTOR through direct binding. Downstream of mTOR, S6 kinase 1 (S6K1), and eukaryotic translation initiation factor 4E-binding protein are the two most studied effectors, which are phosphorylated by mTORC1 but not by mTORC2 [11]. Phosphorylation of these two effectors, particularly S6K1 phosphorylation at Thr^389^, has been widely used to detect mTORC1 activity [12].

mTOR is ubiquitously expressed in various tissues and cell types, including undifferentiated and differentiated ESCs. An essential role for mTOR in cell growth and proliferation was demonstrated in both early mouse embryos and ESCs by gene knockout technology [13]. Disruption of mTOR’s kinase domain and treatment with rapamycin both resulted in decreased proliferation of mouse ESCs (mESCs). The specific knockout of *mTor* in the heart, mediated by α*-Mhc* CRE, leads to cardiac dilation and dysfunction [13]. Deletion of *Rheb1*, an upstream molecule of mTORC1, induces impaired heart growth and heart failure [14]. Nonetheless, the roles of rapamycin in cardiac differentiation are still unclear.

In the present study, we evaluated the effect of mTOR inhibition by rapamycin on cardiac differentiation of mESCs and discovered that treatment with rapamycin markedly accelerated the production of spontaneously beating cardiomyocytes triggered by ascorbic acid (AA). Rapamycin promoted the expression of mesodermal and cardiac transcriptional factors. Rapamycin not only caused persistent inhibition of mTOR signaling, but also influenced a host of signaling pathways, including Wnt, Notch1, FGF8 and FGF10 in cardiac differentiation. Our study demonstrated that the combination of rapamycin and AA is an excellent inducer to efficiently generate cardiomyocytes from mESCs.

## Materials and methods

### Cell culture

mESCs (R1) were kindly provided by Dr Huang Tian Yang (Shanghai Jiao Tong University School of Medicine, Shanghai, China). R1 cells were maintained in high glucose DMEM (Gibco) with 15% fetal serum (Vian-saga), 1000 units leukemia inhibitory factor (LIF) (Millipore, Bedford, MA, U.S.A.), 100 mM β-mercaptoethanol (Sigma), 2 mM non-essential amino acids (Gibco. Inc), 100 units/ml penicillin, and 100 mg/ml streptomycin (Sigma–Aldrich, St. Louis, MO, U.S.A.) without feeder cells. Colonies were dissociated using trypsin and passaged at a 1:3 split ratio every 3 4 days depending on the cell density. 293T cells were maintained in high glucose DMEM (Gibco) with 10% fetal serum (Vian-saga), 100 units/ml penicillin, and 100 mg/ml streptomycin.

### Reagents

Rapamycin, Ku0063794, and DMSO were purchased from Sigma.

### Induction and differentiation

To generate embryonic bodies (EBs), cells were digested and dissociated with 0.05% trypsin, and 6 × 10^4^ cells (day 0) were diluted in inducing media (high-glucose DMEM with 20% fetal serum, 100 mM β-mercaptoethanol, 10^−4^ M AA, 2 mM non-essential amino acids), and resuspended in Petri dishes (Jing Dian, Qingdao, China) for 5 days. On day 5, EBs were translocated to cell culture dishes. The medium was refreshed every other day. The EBs started beating on days 7–9, and the rates of beating EBs peaked at day 12.

### Real-time RT-PCR

RNA extractions were performed using TRIzol reagent (Invitrogen) in accordance with the manufacturer’s instructions. For real-time RT-PCR, Thermo sequence detector and SYBR® Green real-time PCR kit (Applied Biosystems, Foster City, CA, USA) were used. The primers used in this study are listed in table 1. Relative PCR signals were normalized to the average expression levels of the undifferentiated samples and normalized ratios were used to indicate up- and down-regulation.

**Table 1:** Primer sequences

Gene name	Accession number	Primer: 5′–3′
18S rRNA	NM_013536.2	F-GTAACCCGTTGAACCCCATT
		R-CCATCCAATCGGTAGTAGCG
*Bmp2*	NM_007553.3	F-TCTTCCGGGAACAGATACAGG
		R-TGGTGTCCAATAGTCTGGTCA
*Bmp4*	NM_001316360.1	F-TTCCTGGTAACCGAATGCTGA
		R-CCTGAATCTCGGCGACTTTTT
*Brachyury T*	NM_009309.2	F-ACCAGAATGAGGAGATTACAGCC
		R-GGAATACCCCGGCTGCTG
*Fgf10*	NM_008002.4	F-TCCGTACAGTGTCCTGGAGATA
		R-GTCATGGGGAGGAAGTGAGC
*Fgf8*	NM_010205.2	F-AGAGCCTGGTGACGGATCA
		R-CTTCCAAAAGTATCGGTCTCCC
*Gata4*	NM_001310610.1	F-CACCCCAATCTCGATATGTTTGA
		R-GGTTGATGCCGTTCATCTTGT
*Hes1*	NM_008235.2	F-ATAGCTCCCGGCATTCCAAG
		R-GCGCGGTATTTCCCCAACA
*Isl1*	NM_021459.4	F-CTGCTTTTCAGCAACTGGTCA
		R-TAGGACTGGCTACCATGCTGT
*Mef2c*	NM_001170537.1	F-CTGAGCGTGCTGTGCGACTGT
		R-GCTCTCGTGCGGCTCGTTGTA
*Mesp1*	NM_008588.2	F-GTCACTCGGTCCTGGTTTAAGC
		R-TGCGTACTGGAACGATGGGT
*Nkx2.5*	NM_008700.2	F-CAAGTGCTCTCCTGCTTTCC
		R-GGCTTTGTCCAGCTCCACT
*Notch1*	NM_008714.3	F-CCCTTGCTCTGCCTAACGC
		R-GGAGTCCTGGCATCGTTGG
*Troponin T2*	NM_001130174.2	F-GGCAGAACCGCCTGGCTGAA
		R-CTGCCACAGCTCCTTGGCCT
*Wnt11*	NM_001285792.1	F-ATGCGTCTACACAACAGTGAAG
		R-GTAGCGGGTCTTGAGGTCAG
α*-Mhc*	NM_001164171.1	F-GCCCAGTACCTCCGAAAGTC
		R-GCCTTAACATACTCCTCCTTGTC
β*-Mhc*	NM_080728.2	F-ACAACCCCTACGATTATGCGT
		R-ACGTCAAAGGCACTATCCGTG
*Tbx5*	NM_011537.3	F-CTCCGTTGAAGCCTTGATCGG
		R-AGACGCCAGGTCAGTGTGA

### Western blot

Cell lysates (50 μg) were prepared and subjected to SDS/PAGE (10% gel) d transferred on to PVDF membranes (Millipore), which were then blocked in 5% non-fat milk for 1 h and incubated with antibodies of target proteins. Antibodies that were used in the present study included Gata4 (1:500; Santa Cruz Biotechnology, Calfonia, U. S. A.), Myosin Heavy Chain antibody MF20 (1:1000; Developmental Studies Hybridoma Bank), p-P70S6K (Ser^389^) (1:1000; Cell Signaling Technology), P70S6K (1:1000; Cell Signaling Technology), ISL1 (1:10 000; Abcam).

### Nuclear extraction

Nuclear extraction experiments were performed as previously described [15]. Specifically, antibodies for target proteins, including lamin B1 (1:1000; Abcam). GAPDH (1:3000; Zhong Shan Golden Bridge co., Beijing, China) and β-catenin (1:500; Santa Cruz Biotechnology, Calfonia, U. S. A.) were used.

### Immunofluorescence

On day 12, cells were digested, cultured on glass coverslips, and fixed in 3.7% (w/v) formaldehyde for 10 min. Indirect immunofluorescent staining for α-actinin (A7811, Sigma) was performed using FITC–conjugated secondary antibody to visualize cellular morphology. Nuclei were counterstained with DAPI (Sigma) for 5 min at room temperature. Immunofluorescence was viewed under an Olympus FV1000 confocal laser scanning microscope.

### FACS analysis

After 12 days of cardiac induction, EBs were collected in 15-ml conical tubes and allowed to settle by gravity. The supernatant was removed and the EBs were washed in PBS. Then, EBs were resuspended in 1 ml 0.05% trypsin-EDTA and incubated at 37ºC for 5 min. Differentiation medium (4 ml) was added to quench the digestion. The dissociated cells were centrifuged (1500 rpm) and the supernatant was removed, 100 µl of cytofix buffer (554714, Becton Dickinson) was added, incubated in the dark at room temperature for 20 min, then washed twice in wash buffer (554723, Becton Dickinson). Cells were then stained with an α-Actinin antibody (Sigma; 1:100 dilution in PBS) overnight. Following washes with wash buffer, cells were incubated with an goat-anti-mouse secondary antibody conjugated to Alexa Fluor 488 (1:200 dilution in PBS) for 1 h in the dark. After additional washes in wash buffer twice, the flow cytometry were done by FACSCalibur Flow Cytometer (Becton Dickinson).

### Statistical analysis

All quantificative data were presented as mean values + S.D. Comparisons between groups were analyzed using Student’s *t* test or ANOVA and the Student–Newman–Kleuss method was used to estimate the level of significance. Differences were considered to be statistically significant at *P<0.05 and **P<0.01.

## Results

### Rapamycin alone is insufficient to initiate cardiomyogenesis

**Figure 1:**
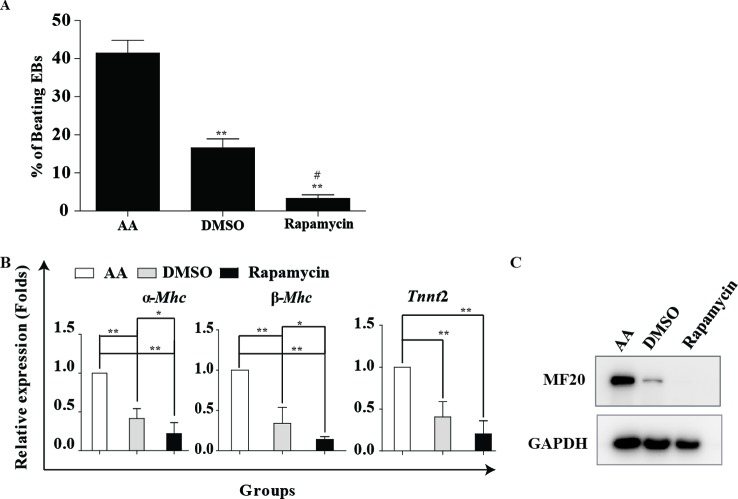
Rapamycin alone is insufficient to initiate cardiomyogenesis. Rapamycin alone is insufficient to initiate cardiomyogenesis. mESCs were induced into cardiomyocytes with 10^−4^ M AA, DMSO (0.1%) and 20 nM rapamycin. Cells were harvested on day 12. (**A**) The number of beating EBs was counted at day 12. Three independent experiments were performed, each in triplicate. **P<0.01 compared with AA, ^#^*P*<0.05 compared with DMSO. (**B**) Real-time PCR analysis of cardiac marker genes α*-Mhc*, β*-Mhc, Tnnt*2 at day 12. Three independent experiments were performed, each in triplicate. *P<0.05, **P<0.01 compared with AA. (**C**) Representative image of Western blot analysis showing MF20 expression. GAPDH is used as an internal control. Three independent experiments were performed.

Rapamycin is an mTOR inhibitor. To determine the effects of rapamycin on cardiac differentiation, the R1 mESCs were exposed to rapamycin or DMSO as vehicle control in suspension culture in medium without LIF. AA, a well-known cardiac differentiation inducer, was used as a positive control for initiating cardiac differentiation or evaluating differentiation efficiency. ESCs aggregated to form EBs in suspension culture; then they were plated for adherent culture at day 5. Generally, spontaneously beating cardiomyocytes could be observed after 7 days of AA treatment. The morphologies of ESCs could be seen in Supplementary Figures S1, S2. When ES cells were induced by 20 nM rapamycin in the absence of AA, only a few spontaneously beating EBs occurred in the rapamycin group (Figure 1A). Furthermore, the cardiac differentiation efficiency of the rapamycin group was lower than the vehicle group. The mRNA level of cardiac structural and contractile proteins α*-Mhc*, β*-Mhc* and *Tnnt*2 and the protein level of MF20 also confirmed the invalid effect of rapamycin alone in cardiac induction (Figure 1B,C). Therefore, these results indicate that rapamycin alone is insufficient to initiate cardiomyogenesis but instead to surpress this progress.

### Rapamycin promotes cardiac differentiation in mESCs with AA

**Figure 2:**
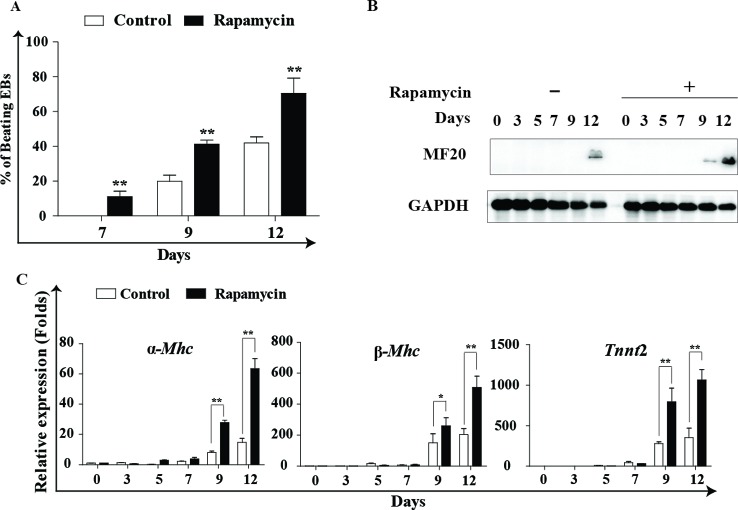
Rapamycin promotes cardiac differentiation from mESCs with AA. Rapamycin promotes cardiac differentiation from mESCs with AA. mESCs were induced into cardiomyocytes using 10^−4^ M AA, and simultaneously the cells were treated with 20 nM rapamycin or DMSO (0.01%) from day 0 to day 12. Cells were harvested at indicated times. Rapamycin: 20 nM rapamycin + AA treated from day 0 to day 12; control: DMSO (0.01%) + AA treated from day 0 to day 12. (**A**) The percentage of contracting EBs was calculated at days 7, 9 and 12. Three independent experiments are shown. *P<0.05, **P<0.01 compared with control. (**B**) Representative image of MF20 Western blot analysis is shown. GAPDH is used as an internal control. Three independent experiments were performed. (**C**) Real-time PCR analysis of cardiac marker genes *α-Mhc*, β*-Mhc, Tnnt*2. Three independent experiments were performed, each in triplicate. *P<0.05, **P<0.01 compared with control.

Our further experiments found that rapamycin combined with AA effectively promoted cardiomyogenesis as evaluated by assessment of the percentage of spontaneously beating embryoids in AA + 0.01% DMSO (control) and AA + rapamycin groups (rapamycin). The maximal differentiation-promoting effect was achieved with 20 nM rapamycin, and the percentage of beating EBs in this group reached 70.42 ± 8.80% compared with 38.44 ± 3.9% in the control group (Figure 2A). The expression analysis of MF20 protein further confirmed the optimal effect of 20 nM rapamycin on cardiomyogenesis (Figure 2 B). Real-time PCR analysis was used to detect the expression of cardiac structural and contractile proteins α*-Mhc*, β*-Mhc* and *Tnnt*2 at indicated time points. Compared with control, cells treated with 20 nM rapamycin showed higher expression of cardiac functional markers (Figure 2C). Additionally, treatment with 20 nM rapamycin did not significantly affect cell viability, mortality, or cell proliferation. High dosages (≥80 nM) of rapamycin favored cardiomyogenesis, but caused evident cell apoptosis and growth arrest (results not shown).

### Rapamycin promotes the expression of mesodermal and cardiac transcription factors

**Figure 3:**
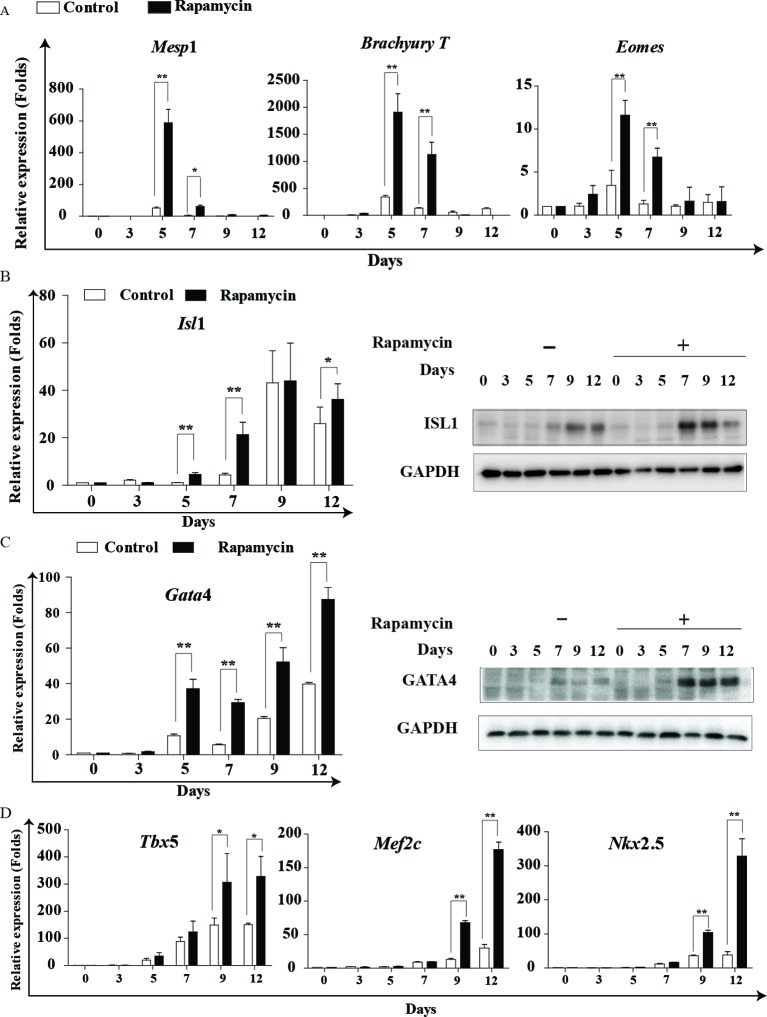
Rapamycin promotes the expression of mesodermal and cardiac transcriptional factors. Rapamycin promotes the expression of mesodermal and cardiac transcriptional factors. (**A**) Real-time PCR analysis of the expression of *Mesp*1, *Brachyury T* and *Eomes*. (**B,C**) Representative images of Western blot analysis and real-time PCR analysis were used to detect the expression of ISL1 and Gata4. (*P<0.05, **P<0.01 compared with control). (**D**) Real-time PCR analysis of cardiac-specific transcription factor markers *Tbx*5, *Mef*2c and *Nkx*2.5. Rapamycin: 20 nM rapamycin + AA treated from day 0 to day 12; control: DMSO (0.01%) + AA treated from day 0 to day 12. Three independent experiments were performed, each in triplicate. Each bar represents mean ± S.D. from three samples (*P<0.05, **P<0.01 compared with control).

Given that stem cells undergo a consecutive array of differentiation to transform into cardiac cells, we employed real-time PCR to illuminate the precise effects of rapamycin at distinct stages of cardiomyogenesis. *Mesp*1, *Brachyury T* and *Eomes* are required for mesoderm formation and maturation [16–19]. As shown in Figure 3A, *Mesp*1, *Brachyury T* and *Eomes* expression sharply increased in the rapamycin group. However, the rapamycin-induced increase in *Mes*p1, *Brachyury T* and *Eomes* at the mRNA level was transient and only occurred at days 5 and 7 of cardiac differentiation. Therefore, our results indicate that rapamycin is beneficial for mesoderm differentiation.

*Isl*1 is one of the earliest markers for the developing cardiac mesoderm, lying genetically upstream of the cardiac progenitor markers *Gata*4, *Nkx*2.5 and *Mef*2*c* [20]. The synergy of *Gata*4, *Nkx*2.5 and *Mef*2*c* evokes the expression of cardiac structural and contractile genes, such as α*-Mhc* and β*-Mhc* [21]. Comparatively, *Isl*1 showed a significant increase at differentiation day 5 (mRNA) and day 7 (protein) in the rapamycin group (Figure 3B), and *Gata*4 exhibited a similar expression pattern (Figure 3C). Other cardiac-specific transcription factor markers, such as *Tbx*5, *Nkx*2.5 and *Mef*2*c*, showed an increase in gene expression after rapamycin treatment (Figure 3D). However, up-regulation of *Tbx*5, *Nkx*2.5 and *Mef*2*c* occurred at day 9. These results suggest that *Isl*1 and *Gata*4 are two major target genes of rapamycin. Given the importance of *Gata*4 in cardiac gene expression, the augmentation of *Tbx*5, *Nkx*2.5, *Mef*2*c* and other cardiac functional genes may be a direct downstream event of *Gata*4 up-regulation. Collectively, our results reveal that rapamycin promotes cardiomyogenesis via facilitating the state conversion of mesoderm and cardiac mesoderm, and that Gata4 is an important modulator mediating the differentiation-promoting effects of rapamycin.

### Rapamycin promotes cardiac differentiation at the early and later stages

**Figure 4:**
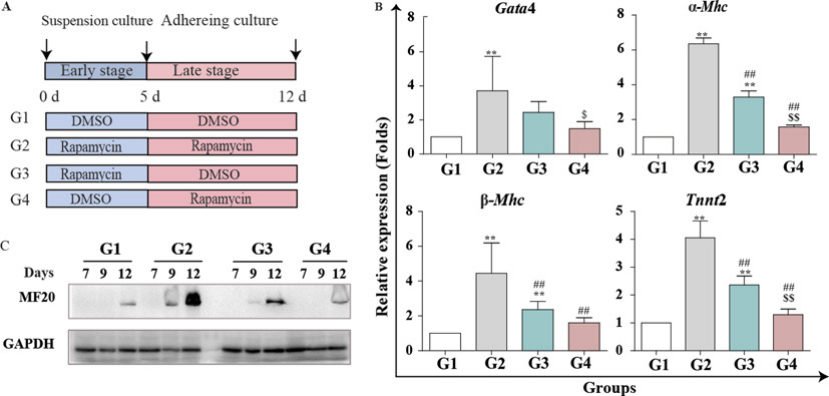
Rapamycin promotes cardiac differentiation at early and late stages. Rapamycin promotes cardiac differentiation at early and late stages. Cells were induced by AA and treated with rapamycin (20 nM) at different stages: rapamycin added at days 0–12 (G2), rapamycin added in days 05 (G3), rapamycin added in days 6–12 (G4) and DMSO (0.01%) was added as a control (G1). (**A**) Schematic diagram of differentiation protocol using rapamycin. (**B**) Real-time PCR analysis of cardiac specific genes: *Gata*4, α*-Mhc*, β*-Mhc* and *Tnnt*2. Three independent experiments are shown, each in triplicate (*P<0.05, **P<0.01 compared with G1; ^#^*P*<0.05, ^##^*P*<0.01 compared with G2; ^$$^*P*<0.01 compared with G3). (**C**) Representative image of MF20 Western blot analysis is shown. GAPDH is used as the internal control. Three independent experiments were performed.

The effect of multiple signaling pathways on cardiomyogenesis exhibits remarkable stage-specific characteristics. For instance, Wnt signaling exerts biphasic effects, functioning either as an agonist or antagonist depending on the early or later stages of cardiac differentiation [22]. To further illuminate the crucial stage of rapamycin in the process of cardiomyogenesis, mESCs were induced into cardiomyocytes by AA and treated with rapamycin at different times, including days 0–12 (group 2; G2), days 0–5 (group 3; G3), days 6–12 (group 4; G4). The AA + 0.1% DMSO induced cells were used as the control (group 1; G1) (Figure 4A). The mRNA contents of cardiac marker genes, *Gata*4, α-*Mhc*, β-*Mhc* and *Tnnt*2, were detected by real-time PCR. As shown in Figure 4B, *Gata*4, α-*Mhc*, β-*Mhc* and *Tnnt*2 expression in G2 was significantly higher than in other groups during the full course of cardiac induction. Thus, persistent treatment with rapamycin could achieve the optimal efficiency of cardiac differentiation. Meanwhile, treatment with rapamycin at the early stage of induction (G3) was more effective than the treatment at the later stages (G4; Figure 4B). At the later time point (G4), rapamycin exerted mild, but positive effects on cardiomyogenesis. Similar conclusions were drawn from the immunoblot analysis of MF20 expression (Figure 4C). Taken together, rapamycin can constantly promote cardiomyogenesis during the process of cardiac induction. Nevertheless, the early stage of cardiac differentiation is more critical than the later stages for rapamycin to promote cardiomyogenesis.

### Rapamycin modulates cardiac signal transduction

**Figure 5:**
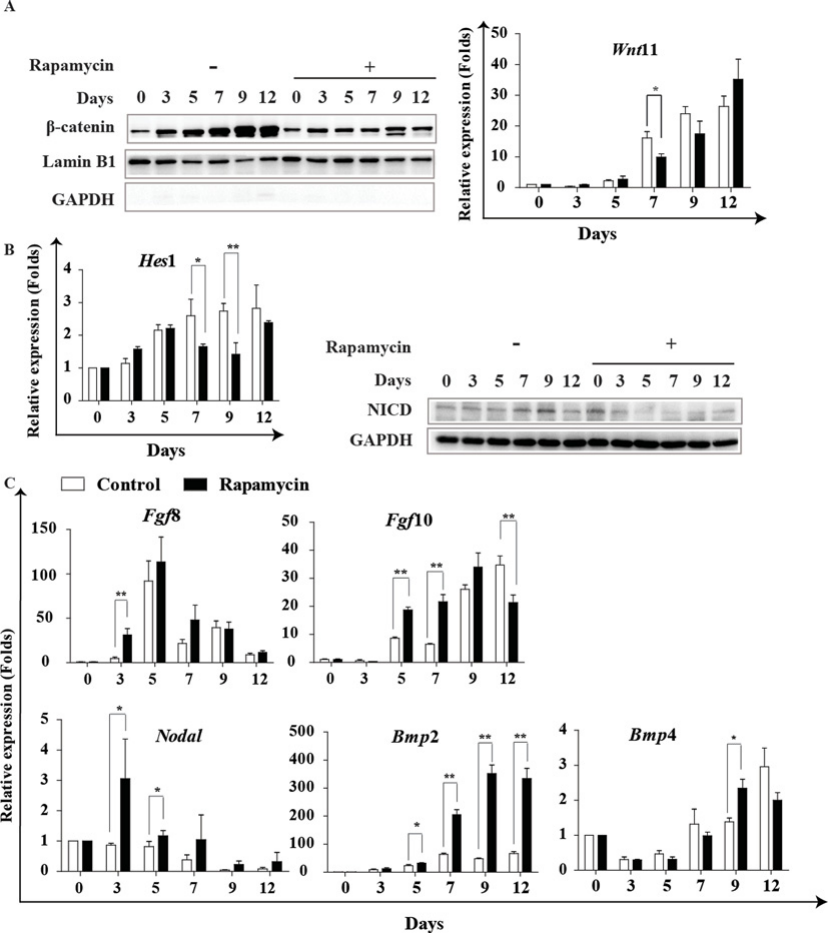
Rapamycin modulates cardiac signal transduction. Rapamycin modulates cardiac signal transduction. (**A**) Representative images of Western blot analysis are shown to detect the expression of β-catenin and the nuclear accumulation of β-catenin. Lamin B1 is used as an internal control for nuclear, and GAPDH is used as an internal control for cytoplasm. Real-time PCR analysis of *Wnt*11 expression at indicated time points. Three independent experiments were performed. (**B**) Real-time PCR analysis of *Hes*1 in indicated time points. Three independent experiments are shown, each in triplicate; each bar represents mean ± S.D. of three samples (*P<0.05, **P<0.01 comapred with control). Representative images of Western blot analysis are shown to detect the activated NICD1 at indicated time points. Three independent experiments were performed. (**C**) Real-time PCR analysis of *Fgf*8, *Fgf*10, *Noda*1, *Bmp*2 and *Bmp*4 expression at indicated time points. Rapamycin: 20 nM rapamycin + AA treated from day 0 to day 12; control: DMSO (0.01%) + AA treated from day 0 to day 12. Three independent experiments are shown, each in triplicate; each bar represents mean ± S.D. from three samples (*P<0.05, **P<0.01 compared with control).

Subsequently, we assessed the effects of rapamycin on signal pathways, such as Wnt, Notch, FGF and BMP, which are important for cardiac differentiation and play distinct roles at different differentiation stages. Western blot analysis showed that rapamycin reduced the nuclear accumulation of β-catenin, especially at the later stages of induction (days 7–9) (Figure 5A left). This result suggests that rapamycin can block the activation of Wnt/β-catenin signaling at the later stage. However, we showed that the expression of *Wnt*11 exhibited no obvious change after rapamycin treatment (Figure 5A right). Furthermore, we found that *Hes*1 expression, the Notch downstream gene, decreased after rapamycin treatment (Figure 5B left). Western blot analysis uncovered that rapamycin decreased the expression of activated Notch1 intracellular domain (NICD1) at the later stages (Figure 5B right), but *Notch* 1 expression was not different (results not shown).

Meanwhile, real-time PCR analysis showed that *Fgf*8 was up-regulated at day 3 and *Fgf*10 was up-regulated at days 5 and 7 under rapamycin treatment compared with control (Figure 5C). Nodal signaling regulates mesoderm specification during cardiac differentiation [23]. Our results showed that *Nodal* expression increased during the early days of differentiation after rapamycin treatment (Figure 5C). When mESCs were treated with rapamycin, *Bmp*2 expression was significantly enhanced during days 7–12 of differentiation, but the promotion of *Bmp*4 was not remarkable (Figure 5C). Taken together, our findings indicate that rapamycin can differently modulate multiple signaling pathways during specific stages of cardiac induction. Rapamycin facilitates the expression of *Fgf*8 and *Nodal* at the early stage, and inhibits the activation of Wnt/β-catenin and Notch but promotes *Bmp2* signaling at the later stage.

### Rapamycin inhibits mTOR signaling to facilitate cardiac differentiation

**Figure 6:**
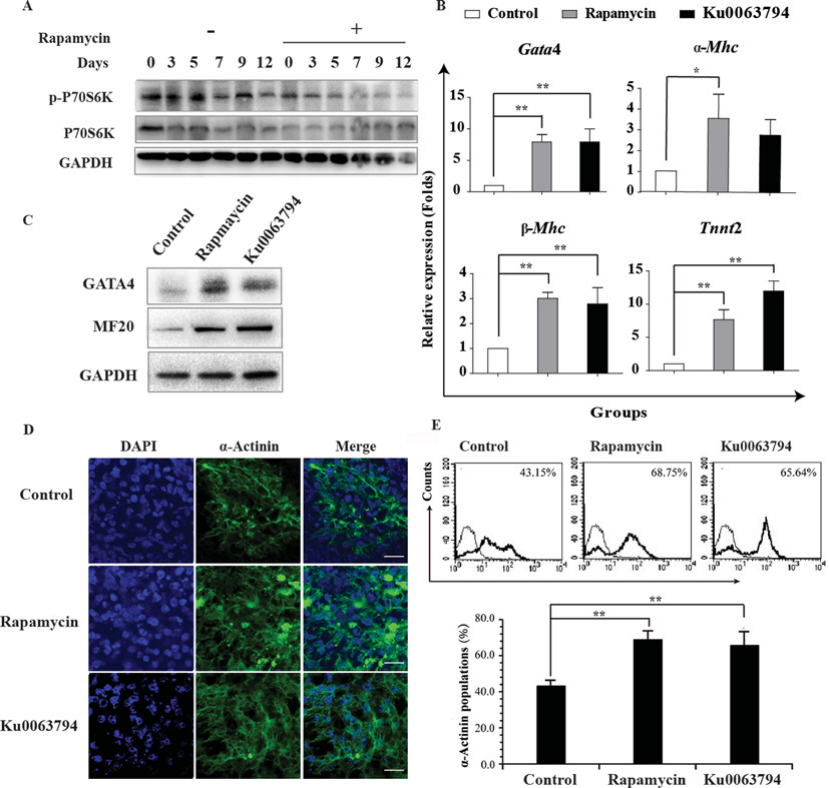
Rapamycin performs its role through inhibition of mTOR pathway. Rapamycin performs its role through inhibition of mTOR pathway. (**A**) Representative images of Western blot analysis are shown, which were used to detect the phosphorylation level of mTORC1 downstream P70S6K at the indicated time points. Three independent experiments were performed. (**B**) Cells were treated with 20 nM rapamycin + AA or 1 μM mTOR kinase inhibitor Ku0063794 + AA from day 0 to day 12. DMSO (0.01%) + AA is used as a negative control. The cells were harvested at day 12 and subsequently analyzed for expression of *Gata*4, α*-Mhc*, β*-Mhc*, and *Tnnt*2 by real-time PCR. Three independent experiments are shown, each in triplicate. Each bar represents mean ± S.D. from three samples (*P<0.05, **P<0.01). (**C**) Representative images of Western blot analysis are shown, which were used to detect the protein expression file of MF20 and GATA4 in cells harvested at day 12. (**D**) Immunofluorescence staining of α-actinin (green) in control, rapamycin and Ku0063794 groups at day 12. Nuclei were counterstained with DAPI (blue). Scale bar 20 μm. (**E**) Representative FACS analysis showing an increase in the fraction of α-actinin^+^ cells following rapamycin or Ku0063794 treatment compared with control. Three independent experiments were performed.

To explore the underlying mechanism of rapamycin-mediated cardiac differentiation, we detected the phosphorylation of P70S6K, which is an mTORC1 downstream member. Phosphorylation of P70S6K at Thr^389^ can reflect mTORC1 activity [11]. As shown in Figure 6A, we observed that P70S6K phosphorylation gradually decreased along with the progress in cardiac differentiation. The protein level of p-P70S6K decreased after rapamycin treatment, implicating the inhibition of mTOR signaling. To exclude the possibility that rapamycin promotes cardiac differentiation via other mechanisms independent on mTOR signaling, we used the mTOR kinase inhibitor Ku0063794 to assess its effects on cardiomyogenesis. Likewise, Ku0063794 enhanced the efficiency of cardiac induction as demonstrated by the expression of cardiac genes *Gata*4, α*-Mhc*, β*-Mhc* and *Tnnt*2 (Figure 6B). MF20 and GATA4 were greatly up-regulated after treatment with rapamycin or Ku0063794 (Figure 6C). Immunostaining and FACS for the cardiac marker α-actinin reveals that cardiac differentiation was enhanced by treatment with rapamycin or Ku0063794 (Figure 6D,E). These results show that the inhibition of mTOR signaling can enhance cardiac differentiation.

## Discussion

Cardiomyogenesis *in vitro* has been illustrated to recapitulate the *in vivo* developmental stages [24]. Stem cells undergo a stepwise committed differentiation from mesoderm, cardiac mesoderm and cardiac progenitors, to functional cardiomyocytes. Cardiomyogenesis is a strictly orchestrated process converging with a set of signaling pathways and transcriptional factors in a synergistic manner. Multiple signaling pathways, such as BMP, Wnt, Notch, Nodal and FGF, play crucial roles in the progressive process of cardiac differentiation. Notably, several signaling pathways exert temporal effects on cardiomyogenesis. For instance, Wnt and Notch pathways have a biphasic role in cardiomyogenesis. They generally promote the mesoderm induction at the early stage, but impose opposite effects on the formation and maturation of cardiac progenitors at the later stages of cardiac differentiation [25,22]. Distinct types of Wnt signaling inhibitors and activators have been verified to promote cardiomyogenesis at specific stages [26–28]. Therefore, the differentiation stage of cardiomyogenesis must be taken into consideration during cardiac induction with specific growth factors or small molecules. Sequential induction imitating the stepwise procedure of cardiac differentiation *in vivo* is practical to produce cardiomyocytes for scientific research and medical application.

In the present study, we provide a simple and convenient protocol for massive generation of cardiomyocytes derived from ESCs with rapamycin treatment. Rapamycin is an mTOR inhibitor and an autophagy inducer. We found that persistent treatment with rapamycin enhanced the differentiation efficiency of mESCs induced by AA. However, rapamycin alone was insufficient to initiate cardiomyogenesis but seemed to suppress it. It was reported that inhibition of mTORC1 by knockdown of raptor promotes the cardiac differentiation [29], but inhibition of mTORC2 via knockdown of its component rictor suppresses cardiac differentiation [30]. It seems that the two complexes have opposite effects on cardiac differentiation. Long-period treatment of rapamycin leads to the inhibition of assembly of mTORC2 [10]. We speculated that rapamycin inhibits both mTORC2 and mTORC1 in our inducing condition. AA was reported to enhance the synthesis of collagen IV to promote cardiac differentiation [31], a series of molecular signals initiated by collagen, such as ERK, JNK, stat1/3 pathway [32], which promote cardiac differentiation [33–35]. Moreover, AA also suppressed mTORC1 [36]. AA may strengthen mTORC1 inhibition co-operating with rapamycin to induce mESC differentiation. Rapamycin can constantly promote cardiomyogenesis throughout the process of cardiac induction. Additionally, rapamycin promotes the cardiac differentiation of the mouse teratoma cells p19cl6 (Supplementary Figures S3), which confirmed the function of rapamycin. Thus, treatment with the combination of rapamycin and AA might be a more simple and effective protocol for cardiac induction.

Though rapamycin promoted cardiac differentiation at both the early and later stages, rapamycin was more effective at the early stage of differentiation. Mechanistic studies have shown that rapamycin promotes the expression of mesodermal and cardiac transcriptional factors. The expression of *Mesp*1, *Brachyury T* and *Eomes*, required for mesoderm formation and maturation, showed a dramatic and transient increase following the rapamycin treatment. Meanwhile, rapamycin enhanced the expression of *Isl*1 and *Gata*4, which are the marker genes of the cardiac mesoderm and cardiac progenitor, respectively. Mutual regulations between Gata4 and other cardiac transcriptional factors exist, such as Nkx2.5 and Mef2c. In addition, *Gata*4 plays vital roles in the transcription regulation of cardiac functional genes, such as α*-Mhc*, β*-Mhc* and *Tnnt*2. Therefore, we assumed that Gata4 is an important modulator in mediating the differentiation-promoting effects of rapamycin.

Additionally, rapamycin exerted versatile effects on multiple signaling pathways associated with cardiac differentiation. Rapamycin enhanced the expression of *Fgf*8 and *Nodal* at the early stage of differentiation. It is well known that *Nodal* signaling governs the differentiation of stem cells towards mesodermal cells. *Fgf*8 might stimulate the mesodermal commitment and expansion of stem cells [37]. At the later stages of cardiac induction, rapamycin effectively inhibited the activation of Wnt/β-catenin and Notch signaling, which are believed to exert inhibitory roles at later stages of differentiation [25,22]. Rapamycin reduced the nuclear accumulation of β-catenin and the abundance of active NICD1. BMP signaling was believed to promote cardiac specification; rapamycin treatment leads to an increase of *Bmp*2. A previous report showed that mTOR signaling inhibits BMP signaling by binding to BMRPII and smad 1, 5, 8 and that rapamycin activates BMP signaling [38], but the mechanism of rapamycin increasing the expression of *Bmp*2 needs further research.

mTOR signaling has been reported to inhibit the differentiation of the mesoderm and endoderm but promotes the differentiation of the ectoderm, and our results correspond with this finding. mTOR exerts its main function by forming two complexes: mTORC1 and mTORC2. Rapamycin and its analogs bind to the cytosolic 12-kDa FK506-binding protein, thereby forming a protein complex that only targets a specific domain of the mTOR protein when it is a part of mTORC1. However, it has been reported that prolonged treatment with rapamycin inhibits mTORC2 assembly [10]. Herein, our results show that mTORC1 was inhibited by rapamycin as the phosphorylation level of p70S6K decreased. The use of the mTOR kinase inhibitor Ku0063794, which inhibits both mTORC1 and mTORC2, showed a similar promotion of cardiogenesis. However, inhibition of mTORC2 resulted in a dramatical decrease in cardiomyocytes differentiation [30] and inhibition of mTORC1 by knockdown of raptor promotes the cardiac differentiation [29]. Therefore, we speculate that rapamycin plays its roles mainly through inhibition of mTORC1.

Overall, our results highlight a potential role of mTOR signaling in cardiac differentiation and provide a simple and convenient protocol for massive generation of cardiomyocytes derived from ESCs using rapamycin treatment.

## Abbreviations

AA: Ascorbic acid
AKT: Protein kinase B
AMPK: AMP-activated protein kinase
BMP: Bone morphogenetic protein
CRE: Cre recombinase
DMEM: Dulbecco’s modiﬁed Eagle's medium
EB: Embryonic body
ERK: Extracellular signal-regulated kinase
ESC: Embryonic stem cell
FGF: Fibroblast growth factor
FK-506: Tacrolimus
GAPDH: Glyceraldehyde-3-phosphate dehydrogenase
GATA4: GATA binding protein 4
ISL1: Insulin gene enhancer binding protein -1
JNK: c-Jun N-terminal kinase
LIF: Leukemia inhibitory factor
Mesp1: Mesoderm posterior 1 homolog
Mef2c: Myocyte-specific enhancer factor 2C
mESC: Mouse embryonic stem cell
MF20: Myosin heavy chain (MHC) antibody, represents MHC
mTOR: Mammalian target of rapamycin
mTORC1: Mammalian target of rapamycin complex 1
mTORC2: Mammalian target of rapamycin complex 2
Nkx2.5: Homeobox protein Nkx-2.5
NICD1: Notch 1 intracelluar domain
Notch: Neurogenic locus notch homolog protein
PI3K: Phosphatidylinositol-4,5-bisphosphate 3-kinase
Rheb: Ras homolog enriched in brain
S6K1: Ribosomal protein S6 kinase beta-1, also known as P70S6K
Smad: Mothers against decapentaplegic homolog
Tbx5: T-box transcription factor TBX5
Tnnt2: Cardiac muscle troponin T
Wnt: The int/Wingless family protein
